# Special Issue: “Host Factors in Plant Viral Infections”

**DOI:** 10.3390/v15010024

**Published:** 2022-12-21

**Authors:** María Amelia Sánchez Pina

**Affiliations:** Plant Pathology Group, Stress Biology and Plant Pathology Department, CEBAS-CSIC, Campus Universitario de Espinardo 25, 30100 Murcia, Spain; spina@cebas.csic.es

I would like to thank all the authors that have published their manuscripts, the scientists who reviewed submitted manuscripts and made suggestions that improved the reports, and the editorial staff workers who put this Special Issue together. I sincerely believe that all articles published here will be of great interest to the readers of *Viruses*.

M. Rocher et al. [1] studied one of the REMORIN proteins belonging to a plant-specific multigene family localized in plasma membrane nanodomains and in plasmodesmata, demonstrating another level of specificity and the complexity of the mode of action of REMORIN proteins during plant virus infection. 

Z. Li et al. [2] studied NSvc4, the movement protein of rice stripe virus (RSV). They showed that chloroplast-localized NSvc4 can impair chloroplast-mediated immunity, and that expressing NSvc4 in *Nicotiana benthamiana* leaves results in the decreased expression of the defense-related genes *NbPR1*, *NbPR2*, and *NbWRKY12* and the inhibition of chloroplast-derived ROS production. 

B. Garcia et al. [3] studied the P1b protein of cucumber vein yellowing virus (CVYV), a type B P1 protein, to try to identify possible factors that could play a relevant role during viral infection. Their characterization of the interaction between P1b and the *N. benthamiana* homolog of Importin 7, and its effect on the plant, identified a new possible role of this host protein as a regulator of the action of viral RNA silencing suppressors.

T.A. Helderman et al. [4] studied host factors that are important for the systemic spread of *Tomato spotted wilt orthotospovirus* (TSWV) and disease symptom development. Their data indicate that silencing one clade of the *eEF1A* gene family strongly suppressed TSWV accumulation and disease symptom development in *N. benthamiana*, suggesting that the gene products act as important susceptibility factors for TSWV.

C. Chen et al. [5] studied the identification of 15 genes of the RAV family in the rice genome. The RAV family is part of the B3 superfamily and is one of the most abundant transcription factor families in plants. Based on domain similarity and phylogenetic topology, rice RAV transcription factors were phylogenetically clustered into four groups. Their results suggest that the rice *RAV* genes are involved in diverse signaling pathways and in varied responses to virus infection.

M. Diop and J.L. Gallois [6] studied phosphoglycerate kinases (PGKs), which have emerged as a new class of susceptibility factors to single-stranded positive RNA viruses, including potyviruses. They assessed the diversity and homology of chloroplastic and cytosolic PGK sequences in several crops and reviewed the current knowledge on their redundancies during plant development using *Arabidopsis* as a model, showing how PGKs are involved in susceptibility—and resistance—to viruses.

V.V. Oberemok et al. [7] studied the “host–virus–insect” triangle that best describes the life cycle of plant ss(+)RNA viruses, where the host is the place of reproduction and the insect is the distributor, and sometimes the place of reproduction as well. In their opinion, ss(+)RNA plant viruses manifest themselves as great opportunists and ecosystem tuners. This is a consequence of their short life cycle, the peculiarities of the functioning of RNA-dependent RNA polymerase, and the minimum number of substances necessary for the reproduction of viral particles.

L. Alvarado-Marchena et al. [8] studied the roles of the two putative m^6^A-sites in the AMV cycle by introducing compensatory point mutations to interfere with or abolish the m^6^A methylation. These results extend the knowledge of the requirement of hpE in the AMV infection cycle, so that both the identity and base-pairing capability of bases in this structure are essential for minus-strand and plus-strand synthesis.

Finally, as Guest Editor, I would like to conclude by saying that identifying new interactions between plant-host factors and viral proteins is a difficult but necessary task, since it is this interplay that reveals the actual functions and mechanisms behind the viral infection. I am confident that the collected articles will inspire more research. There is still much research to be done on this interesting topic, and I encourage all colleagues working on plant viruses to dedicate a part of their work to a particular plant–virus interaction to address this topic.

## References

[1] Rocher M., Simon V., Jolivet M.-D., Sofer L., Deroubaix A.-F., Germain V., Mongrand S., German-Retana S. (2022). StREM1.3 REMORIN Protein Plays an Agonistic Role in Potyvirus Cell-to-Cell Movement in *N. benthamiana*. Viruses.

[2] Li Z., Li C., Fu S., Liu Y., Xu Y., Wu J., Wang Y., Zhou X. (2022). NSvc4 Encoded by Rice Stripe Virus Targets Host Chloroplasts to Suppress Chloroplast-Mediated Defense. Viruses.

[3] García B., Bedoya L., García J.A., Rodamilans B. (2021). An Importin-β-like Protein from *Nicotiana benthamiana* Interacts with the RNA Silencing Suppressor P1b of the Cucumber Vein Yellowing Virus, Modulating Its Activity. Viruses.

[4] Helderman T.A., Deurhof L., Bertran A., Boeren S., Fokkens L., Kormelink R., Joosten M.H.A.J., Prins M., van den Burg H.A. (2021). An Isoform of the Eukaryotic Translation Elongation Factor 1A (eEF1a) Acts as a Pro-Viral Factor Required for Tomato Spotted Wilt Virus Disease in *Nicotiana benthamiana*. Viruses.

[5] Chen C., Li Y., Zhang H., Ma Q., Wei Z., Chen J., Sun Z. (2021). Genome-Wide Analysis of the RAV Transcription Factor Genes in Rice Reveals Their Response Patterns to Hormones and Virus Infection. Viruses.

[6] Diop M., Gallois J.-L. (2022). Exploring New Routes for Genetic Resistances to Potyviruses: The Case of the *Arabidopsis thaliana* Phosphoglycerates Kinases (PGK) Metabolic Enzymes. Viruses.

[7] Oberemok V.V., Puzanova Y.V., Kubyshkin A.V., Kamenetsky-Goldstein R. (2021). Top Three Strategies of ss(+)RNA Plant Viruses: Great Opportunists and Ecosystem Tuners with a Small Genome. Viruses.

[8] Alvarado-Marchena L., Martínez-Pérez M., Úbeda J.R., Pallas V., Aparicio F. (2022). Impact of the Potential m^6^A Modification Sites at the 3′UTR of Alfalfa Mosaic Virus RNA3 in the Viral Infection. Viruses.

